# ChatGPT in forensic sciences: a new Pandora’s box with advantages and challenges to pay attention

**DOI:** 10.1093/fsr/owad039

**Published:** 2023-11-10

**Authors:** Ricardo J Dinis-Oliveira, Rui M S Azevedo

**Affiliations:** 1H-TOXRUN—One Health Toxicology Research Unit, University Institute of Health Sciences (IUCS-CESPU), CESPU, CRL, Gandra, Portugal; Department of Public Health and Forensic Sciences and Medical Education, Faculty of Medicine, University of Porto, Porto, Portugal; UCIBIO/REQUIMTE, Laboratory of Toxicology, Faculty of Pharmacy, University of Porto, R. Jorge Viterbo Ferreira, n° 33-A, Lisboa, Portugal; FOREN—Forensic Science Experts, Dr. Mário Moutinho Avenue, n.° 33-A, Lisbon, Portugal; 1H-TOXRUN—One Health Toxicology Research Unit, University Institute of Health Sciences (IUCS-CESPU), CESPU, CRL, Gandra, Portugal

**Keywords:** ChatGPT, artificial intelligence, forensic sciences, medicine, advantages and limitations

## Abstract

ChatGPT is a variant of the generative pre-trained transformer (GPT) language model that uses large amounts of text-based training data and a transformer architecture to generate human-like text adjusted to the received prompts. ChatGPT presents several advantages in forensic sciences, namely, constituting a virtual assistant to aid lawyers, judges, and victims in managing and interpreting forensic expert data. But what would happen if ChatGPT began to be used to produce forensic expertise reports? Despite its potential applications, the use of ChatGPT and other Large Language Models and artificial intelligence tools in forensic writing also poses ethical and legal concerns, which are discussed in this perspective together with some expected future perspectives.

## Introduction

ChatGPT is one of the largest publicly advanced language models that use deep learning techniques to produce human-like responses to natural language prompts [1]. It is part of the family of generative pre-training transformer (GPT) models developed by OpenAI. Following an eloquent and pleasant-to-read vocabulary and using a vast corpus of text data, ChatGPT can “capture the nuances and intricacies of human language, allowing it to generate appropriate and contextually relevant responses across a broad spectrum of prompts” [2]. Currently, GPT-4 is the most advanced artificial intelligence (AI) system that was publicly released as of March 2023, with other improved versions already in the works. ChatGPT can be used in a variety of ways, such as chatbots that can carry on conversation with users in a natural and human-like manner, text generation useful for simple or advanced teaching/learning, technical data analysis, programming, business tasks, writing, language translations, etc. Aiming answering questions or performing a specific task, prompts are not restricted to English language and can contain inputs such as spreadsheets, technical specifications, research papers, mathematical equations, etc. [3], with foreseeable multimodal capabilities for audio/video. Despite the long-standing use of AI in various fields such as healthcare systems, its implementation in forensic sciences is almost absent. To interact, usually the human starts a “session” by entering a query (usually referred to as a “prompt”) in plain natural language and receives a rapid “response” that is relevant to the prompt. These exchanges of prompts and responses continue throughout the session like a friendly conversation between two people.

With unprecedented and powerful capabilities for medicine, ChatGPT can help vectorize relevant research fields to invest and help health professionals diagnose and choose the most efficient drug treatments [4]. Specifically, in forensic sciences, ChatGPT also poses a new world of opportunities in this domain by improving the time efficiency and accuracy of judicial decisions. The first steps were recently given in a Preprint that assessed the impact of ChatGPT on digital forensics [5].

Our work complies and updates the advantages, limitations, privacy and ethical concerns, future perspectives, and practical applications of ChatGPT and AI in forensic sciences. We do not expect that AI and ChatGPT will soon replace the need for forensic experts, but those forensic experts who do not use their potential will be outpaced.

## Methods

An exhaustive search was conducted in a range of databases to achieve cross-disciplinary coverage, including PubMed (U.S. National Library of Medicine), Web of Science, Embase and Scopus and Google Scholar, and *via* Google to identify grey literature (those produced outside the traditional commercial/academic publishing channels). Date limit was not applied, but most publications retrieved were released after 2022 since represent the beginning of accessibility of ChatGPT to general population. Multiple combinations of the following keywords were used: ChatGPT, forensic sciences, legal medicine, law, and justice, medicine, drug research, advantages, and limitations. Retrieved journal articles, as well as books, general newspapers, and government documents, were also reviewed for possible additional publications related to this topic. Noteworthy the almost completely absent discussion of ChatGPT in the field of forensic sciences.

### Potential applications of ChatGPT in forensic sciences

The potential applications of ChatGPT in the forensic sciences range from drafting reports to courts and therefore reducing times for judicial decisions, passing by assisting in identifying potential research topics to forensic research groups [6] until produced research papers, including all the involved analyses [7]. Indeed, some studies demonstrates that ChatGPT can produce formal research articles [8] bypassing traditional plagiarism detection methods with median originality score of 100%. Even the AI-output checker identified only 66% of the generated abstracts [9], some authors claim it might be mathematically improbable to be able to do so [10]. Moreover, ChatGPT can potentially be used as a search engine that replies to queries directly rather than directing to sites where the answers must be obtained by the user. It may reduce the time spent by the authors in the daunting process of searching for articles and applying various selection criteria to choose the articles best suiting their forensic topic, and, in turn, allowing them to allocate this time on their actual research work and methodology.

ChatGPT can also lead to the development of virtual forensic assistants to aid victims, lawyers, and judges in managing their forensic and legal data and identify possible matches for uncommon and exceptional difficult cases. In medicine, it has been suggested that it can be utilized to generate automated clinical records, summarizing key data, such as symptoms, adverse effects, imaging and laboratory diagnoses, drug interactions, communication improvements between patients/doctors, economic improvement, treatments, self-diagnosis, and rare diseases identification [11, 12].

In a different perspective, it was found that there is a potential for using ChatGPT as an educational assistance tool to promote interest in education and self-directed learning, particularly in non-face-to-face situations as COVID-19 pandemic [13]. As already suggested for healthcare systems, the utilization of AI might be crucial and imperative because of its ability to enhance precision and accuracy whilst reducing the time required for various aspects of the system.

**Figure 1:**
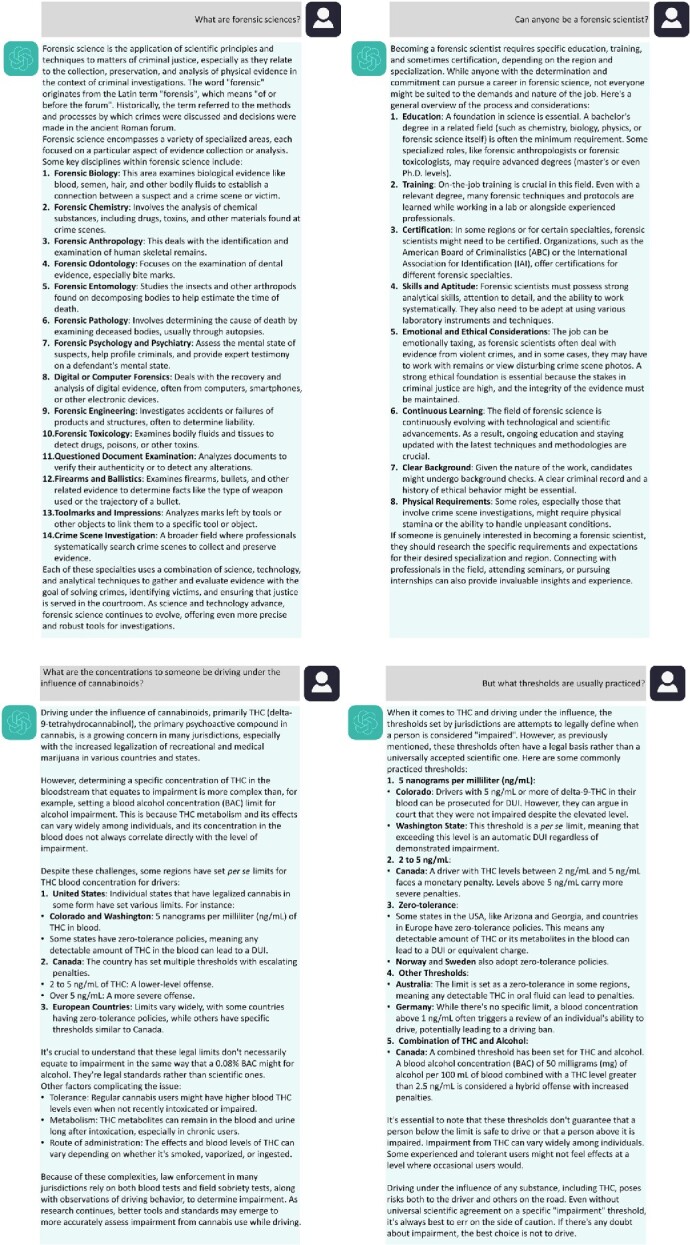
A simple conversation with ChatGPT-4.0 in the field of forensic sciences.

Figure 1 transcripts a typical session with ChatGPT chatbot with three prompts. In comparison to personal previous publications where it was tried to answer the same questions, we did not find relevant incongruencies or major irreconcilable outcomes [14–16]. Moreover, besides the free ChatGPT version 3.5 updated in September 2021, we also used the payable version 4.0. Although more completed responses were registered for the most recent version, major differences were not registered, and therefore, Figure 1 only compiles responses for version 4.0. The main applications, advantages, limitations, and ethical concerns of ChatGPT in forensic sciences are compiled in Table 1.

**Table 1:** Main advantages and limitations of ChatGPT in forensic sciences.

**No**	**Advantages**
1	Personal forensic consulting for victims, lawyers, and judges
2	Personal assistant for forensic education and training
3	Search and analysis current literature (limited to the version updated time)
4	Produces text that avoids conventional plagiarism detection tools
5	Helping in defining the forensic analysis to be done
6	Produces structured text with eloquent vocabulary
7	Increasing forensic literacy and communication between the different judicial and judiciary players
8	Assisting research processes related to forensic sciences (data analysis and processing)
8	Translation
9	Uncovering critical evidence (e.g. datamining) for unsolved cases or offer further insights to innocence projects-related cases
10	Perform different tasks such as “please read and summarize this forensic article”
**No**	**Limitations**
1	Delay in incorporating updates and new developments
2	May raises issues related to the copyright laws
3	Tendency to provide inaccurate responses and repeat phrases from previous interactions
4	Skilled forensic experts are needed to create appropriate prompts and analyze responses in order to differentiate fact from fiction
5	May lead to an overestimation of the truth by judges who believe in the accuracy of AI
6	Unable to distinguish between reliable and unreliable forensic sources as humans
7	Data for AI systems are currently obtained from open sources on the internet, such as open access articles, free books or their extracts, websites, blogs, etc.
8	Data presented in subscription journals, medical records, forensic reports, or judicial decisions only available in a private network are generally excluded

### Challenges and limitations of ChatGPT in forensic sciences

Besides the expected applications, the use of ChatGPT and other AI tools come with several limitations, legal and ethical considerations [17] like credibility [7], plagiarism [18, 19], authorship of ChatGPT in research paper, possible infringement of copyright laws, medico-legal complications, and the potential for inaccuracies or prejudices in the generated content. For instance, the unethical utilization of AI technology may be extended to the hypothesis of fabricate images for research articles (i.e. a type of scientific misconduct) and for court creating new evidence that may bias the judicial decision. This is particularly important since we should be aware of the “seductive allure” potential that images (e.g., neuroimaging) exerts on courts decisions. Indeed, it was demonstrated that juries and judges apparently tend to overestimate the accuracy of neuroscientific evidence [20]. Besides ethical research concerns, in the academy, students can use AI tools to write their homework [21]. In addition, the usefulness in research is questionable, as AI technology may only replicate what has already been done without adding human-like scientific insights. As a result, some scientists oppose using chatbots for research [7]. Thus, those who employ language models in their use must acknowledge these limitations and take measures to guarantee the precision and reliability of the written materials [18]. Biassed court decisions taken by AI-based algorithms should also be highlighted since self-learning algorithms, for instance, may be trained by certain data sets (e.g. tables of toxic drugs, previous decisions, facial images, etc.) that may contain biassed data.

Finally, it should be emphasized that, at present, language models like ChatGPT are not capable of completely taking over the role of human writers, as they lack a similar level of comprehension and specialized knowledge. “Hallucination” is the word typically used to refer to a false response of ChatGPT and that can be particularly dangerous if not properly detected by certified forensic experts [15]. Education is more important than ever.

## Conclusions and future perspectives

Computers and AI have been almost always together since the beginning of their development in the 1940s and 1950s [22]. Following a trajectory of increased performance, speed, and storage and particularly in the field of healthcare, soon it was recognized that computers and AI could be a powerful extension of physician’s intellect either by reading images, predicting drug interactions and high-risk patients [23]. And nowadays chatbot technology is almost everywhere from customer service to personal virtual assistants. Until now, one relevant difficulty in implementing ChatGPT or analogues in forensic sciences is related to know whether the answers provided are grounded in appropriate facts [3]. As already suggested for medicine [23], the onus would be on forensic experts to proofread the work of the chatbot, just as they already do for their forensic reports and exams. In other words, an additional skill is expected from forensic experts, namely, their capacity to answer and proofread. This is even more important when these chatbots propose forensic interpretations and therefore influence judicial decisions as they presently perform diagnoses or recommend treatments for medicine. And several fields of forensic sciences, such as forensic imaging, crime scene analysis, forensic medicine, toxicology (tested in Figure 1), linguistics, fingerprint analysis, anthropology, facial recognition, genetics, estimation of “post-mortem” interval, amongst others, can be revolted [24–26].

**Figure 2:**
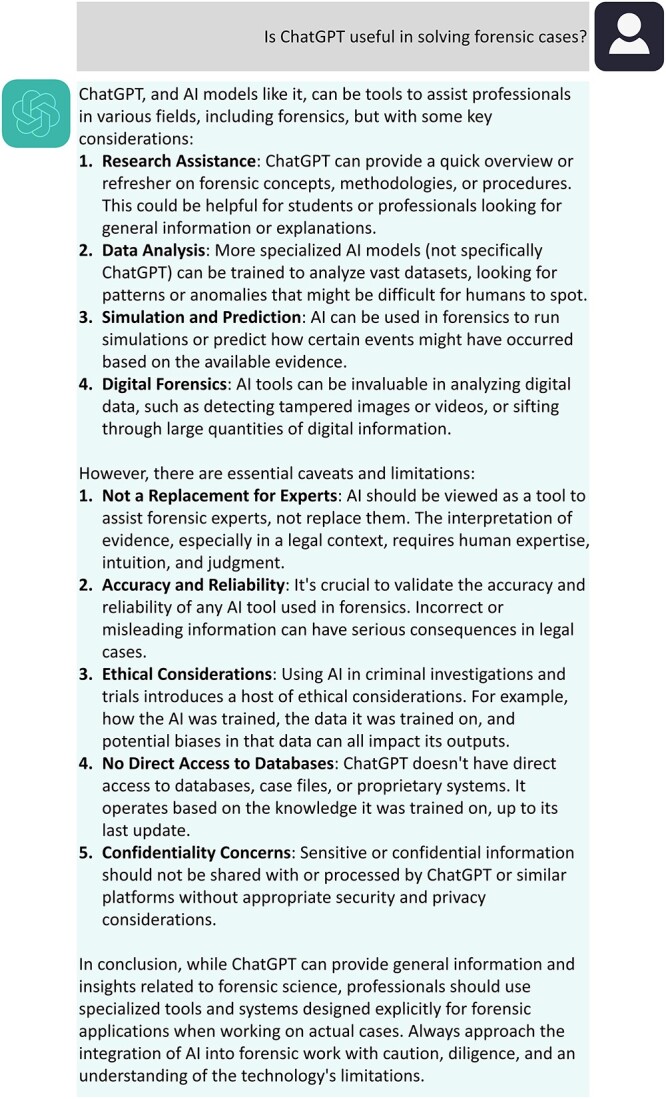
A simple conversation with ChatGPT-4.0 about its use in forensic sciences.

Despite some legitimate concerns, the future of forensic sciences together with ChatGPT is almost beginning and it will not take much time to be an effective assistant. Probably like any other technology its correct utilization can help to solve problems and several universities are already incorporating this technology in the routine education. AI will certainly transform forensic sciences leaving more time for human–human interactions. Moreover, as future AI systems are likely to be far less sensitive to the precise language used in a prompt, less constraints and more accurate responses are expected. In addition, GPT-4.0 and its predecessor GPT-3.5 were not trained specifically for forensic applications, which opens the opportunity to create specific AI platforms for forensic area, increasingly easier with open sourced technologies such as LLAMA2 (https://ai.meta.com/llama/) amongst others. As a future perspective, we asked ChatGPT-4.0 the following: is ChatGPT useful in solving forensic cases? Please note that this question was only made after writing this article and it is curious that ChatGPT also expects the same outcomes and limitations as we discussed. Its fully answer is presented in Figure 2. ChatGPT-4.0 and related future updates and other AI technologies can significantly shape the future of forensic sciences and the fate of judicial decisions by analyzing and uncovering higher probabilistic patterns from large and complex data sets.
